# A physiologically-based flow network model for hepatic drug elimination II: variable lattice lobule models

**DOI:** 10.1186/1742-4682-10-53

**Published:** 2013-09-05

**Authors:** Vahid Rezania, Rebeccah Marsh, Dennis Coombe, Jack Tuszynski

**Affiliations:** 1 Department of Physical Sciences, Grant MacEwan University, Edmonton, AB T5J 4S2, Canada; 2 Department of Physics, University of Alberta, Edmonton, AB T6G 2J1, Canada; 3 Computer Modelling Group Ltd, Calgary, AB T2L 2A6, Canada; 4 Department of Physics and Experimental Oncology, University of Alberta, Edmonton, AB T6G 2J1, Canada

## Abstract

We extend a physiologically-based lattice model for the transport and metabolism of drugs in the liver lobule (liver functional unit) to consider structural and spatial variability. We compare predicted drug concentration levels observed exiting the lobule with their detailed distribution inside the lobule, and indicate the role that structural variation has on these results. Liver zonation and its role on drug metabolism represent another aspect of structural inhomogeneity that we consider here. Since various liver diseases can be thought to produce such structural variations, our analysis gives insight into the role of disease on liver function and performance. These conclusions are based on the dominant role of convection in well-vascularized tissue with a given structure.

## Background

The liver is the major organ responsible for the metabolism and detoxification of drugs. Within the liver, it is the hepatocytes which express a high level of drug-metabolizing enzymes and are primarily responsible for liver drug disposition. Drug access to hepatocytes is governed by transport processes in the well-vascularized liver tissue, and structural variability can obviously impact such transport. In this paper, we extend our physiologically-based lattice model for the transport and metabolism of drugs in the functional unit of the liver [1] (paper I) to consider spatial and structural variability and its impact on hepatic drug metabolism.

### The liver lobule and drug kinetics

#### Functional unit as a regular lattice model

The functional unit of the liver is termed the liver lobule [2] and is the smallest structural unit with complete hepatic functionality. Although various definitions of this unit have been proposed, we have chosen a symmetry element connecting the portal (arterial) tract with hepatic venules. In our first paper, we defined a base-case regular lattice structural model and explored the dynamics of competing convective, diffusive, and reactive processes acting on an injected drug (paclitaxol). Such simulations had the useful consequence of relating drug concentration levels found exiting the lobule to their detailed spatial distribution within the lobule, caused by competing processes. Tables one to four in our first paper detail the parameters chosen for our regular lattice model of the lobule. They provide a basis, and a point of contrast for the drug distributions obtained when some of these assumptions on lobule structure are relaxed, as discussed in this current paper.

### Structural variability

Structural variability of lobule units is expected to be the rule, even among the approximately 1500 lobules that make up the liver of one healthy human. Teutsch and colleagues [4] illustrate this aspect morphologically.

Diseased states can be expected to add a spectrum of additionally variability. The health of the liver can be compromised by viruses, hereditary diseases, and toxins such as alcohol [5]. Damage or death of the hepatocytes leads to inflammation of the liver, called hepatitis. Although zones of necrosis can form when adjacent cells die, this damage is to some extent reversible, since the liver has the ability to regenerate. Thus hepatitis is typically characterized by waves of cell death and regeneration, leading to a mixture of necrotic areas and nodules of new hepatocytes. Because the architecture of the liver is often compromised, some cells may not receive normal levels of blood supply. Furthermore, as inflammation progresses, fibrous tissue may replace the normal hepatocytes, resulting in the irreversible condition of cirrhosis. The damage can be compounded because the formation of necrotic zones increases the resistance to blood flow, and intra-hepatic shunts can occur in which blood vessels begin to bypass the liver altogether. Therefore, although the liver has the capacity to withstand and even correct a lot of damage, its ability to transport, absorb, and metabolize important nutrients and drug molecules can be compromised.

A Base case model, of necessity, required numerous assumptions on an appropriate idealization of the liver lobule structure. In our analysis, we will utilize the concept of a random permeability lattice to capture small vascular irregularities in healthy livers, and the more extreme effects of hypertension sustained due to hepatitis. Damage and scarring occurring with cirrhosis will be treated within the framework of percolation theory, such that the architecture of the liver is physically changed.

### Zonation and elimination kinetics

Zonation is a well-known feature of many metabolic processes occurring in the liver lobule [6], including carbohydrate [7] and nitrogen [8] metabolism, such that some processes are up-regulated near the periportal zone while others are up-regulated near the perivenal side of the lobule. Drug metabolism and drug metabolizing enzymes also show similar zonation features [9]. Our focus will be on the distribution of the cytochrome P450 (CYP) enzyme in particular.

Zonation has been attributed primarily to non-uniform distribution of O_2_ across the lobule [10], with the periportal zone experiencing relatively high concentrations of O_2_ while the perivenal zones see near hypoxic levels of O_2_. The distribution of other factors (e.g. growth factors) has also been shown to play a role. Indeed, injection of specific xenobiotic factors has been utilized to alter zoned enzyme expression levels [11-13]. A future application of our model might be to track O_2_ distribution and metabolism across the lobule. Here, for such a small molecule, molecular diffusion can be expected to play a dominant role.

Following our earlier work, drug uptake and elimination (i.e. conversion to metabolized product) is viewed as a single-step saturable process following Michaelis-Menten kinetics [1].

## Model and methods

**Figure 1:**
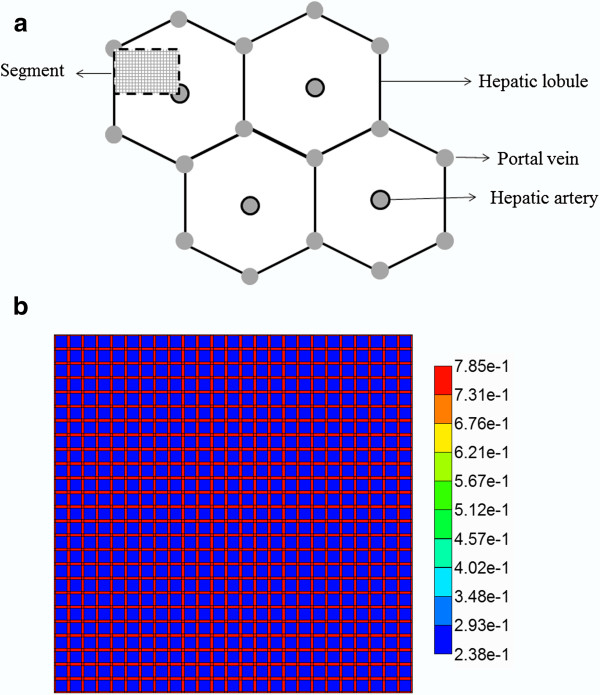
**(a) Schematic diagram of a cross section of hepatic parenchyma consisting of hexagonal lobules, and the portal and hepatic veins.** The lobule contains liver cells called hepatocytes and blood vessels called sinusoids. The segment represents a typical area studied in this paper. **(b)** Homogeneous lattice (segmented area). The high porosity bands represent sinusoids and the lower porosity regions represent tissue containing hepatocytes. The Base case lattice as shown here has tissue porosity 0.2382. The ideal case lattice (not shown) has tissue porosity 0.4764. The extracellular matrix (ECM) case lattice (not shown) has tissue porosity 0.1191.

The flow equations describing reactive-convective-diffusive flow in the liver lobule remain unchanged from our first paper. Figures 1a, b illustrate the network structure of our Base case model from this paper. Here we focus on spatial variability in the flow and reactive parameters.

MATLAB [14] was used to generate multiple realizations of spatial sinusoid permeability variations drawn from a selected sinusoid permeability distribution function (uniform, normal, or log-normal). We are interested in the sensitivity to the spread (i.e. variation) of the distribution of this function, and we will use the term “Random Lattice” to signify our studies in this portion of the work.

Specifically, random permeabilities between [*a*, *b*] (=*K*_sin_ [1-σ, 1 + σ] in our case) was produced by the uniform random generator in MATLAB. Every representation is created independently, multiplied by the sinusoidal permeability *K*_sin_ and then averaged over the number of representations. Based on the central limit theorem, the average value over the sample size *N* approaches the mean value (*a + b*)/2 (=*K*_sin_ in our case) with standard deviation ~ 1/*N*^1/2^( ~ σ /*N*^1/2^ in our case). This means the mean value is independent of σ. Permeability of tissue sites was set to constant *K*_tis_ = 7.35e-2 μm^2^ after the sinusoidal permeability was set and averaged over the number of representations.

**Figure 2:**
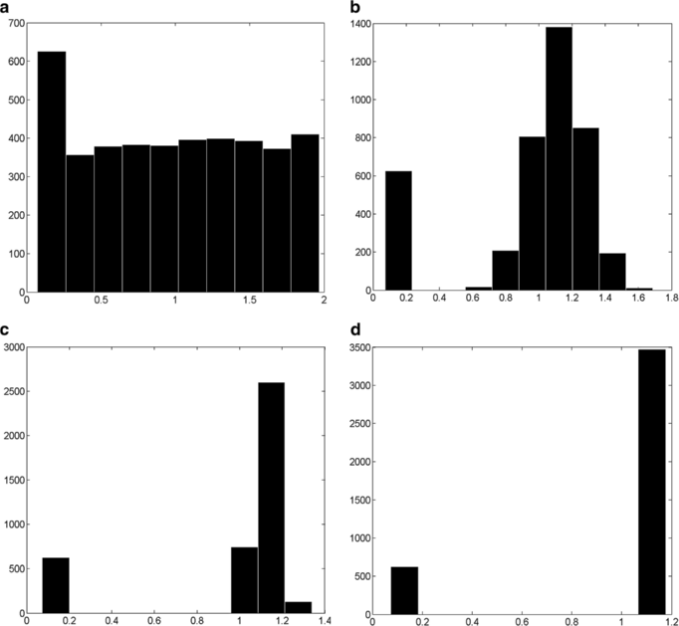
**Random lattice histogram of permeability values for tissue with a fixed sinusoidal values of **${\bar{K}}_{\sin}$**=1.125 μm**^**2 **^**and σ = 0.75. (a)** N = 1 realization, **(b)** N = 10 realizations, **(c)** N = 100 realizations, and **(d)** N = 1000 realizations. The horizontal axis is in μm^2^.

Figures 2a-d show the histogram of permeability values for hepatocyte tissue and sinusoids with *K*_sin_ = 1.125 μm^2^ and σ = 0.75 for 1, 10, 100 and 1000 realizations, respectively. The first peak represents a permeability of tissue value *K*_tis_ = 7.35e-2 μm^2^. As expected, uniform distributed numbers tend toward the mean value. After *N* = 1000 sampling, only two peaks are left, one for the tissue and one for the sinusoids, similar to the fixed tissue-sinusoid permeability case.

Multiple realizations of sinusoidal connectivity and degree of “disconnectiveness” were also generated in a similar manner. Here, the approach to a percolation limit was used to model blockage of flow. Lattices constructed in this manner are termed “Percolation Lattices”. For this case, a random number between [0, 1] was assigned to each site, *X*_i_. Then by comparing the value at each site with a given percolation probability P_c_, the value of that site will change as follows:

$$X_{i}=K_{\sin}\;\mathrm{if}\;X_{i}<=P_{c}\cdot$$

$$X_{i}=0\;\mathrm{if}\;X_{i}>=P_{c}\cdot$$

All X_i_ values were stored in a separate matrix for averaging purpose. Then a new set random number was chosen for each site and the above procedure was repeated up to desired number of samples. At the end, an averaged value found over all representations was calculated for each site. The permeability of tissue sites were again set to the constant 7.35e-2 μm^2^ and the permeability of corner sinusoidal sites set to *K*_sin_.

**Figure 3:**
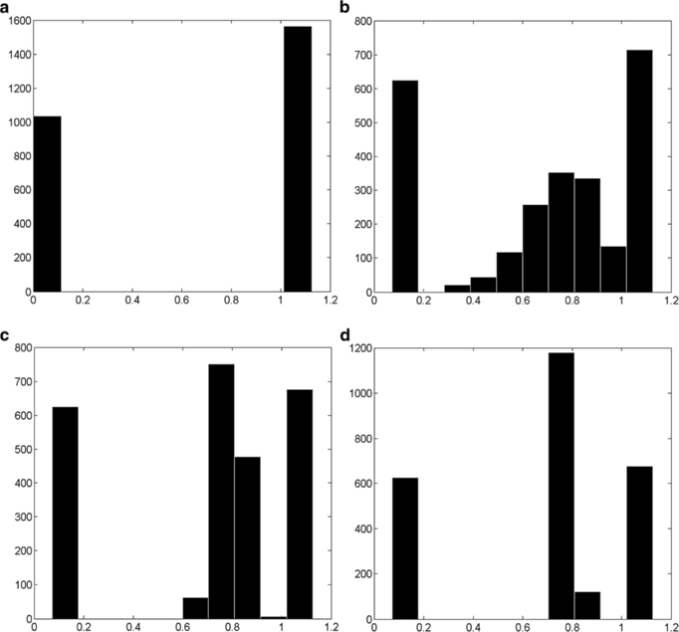
**Percolation lattice histogram of permeability values for tissue with a fixed sinusoidal values of**${\bar{K}}_{\sin}$**=1.125** **μm**^**2 **^**and P**_**c**_ **= 0.7. (a)** N = 1 realization, **(b)** N = 10 realizations, **(c)** N = 100 realizations, **(d)** N = 1000 realizations. The horizontal axis is in μm^2^.

Figures 3a-d show the histogram of permeability values for tissue and sinusoids with *K*_sin_ = 1.125 μm^2^ and *P*_c_ = 0.7 for 1, 10, 100 and 1000 realizations, respectively. The first peak represents a permeability of tissue value of *K*_tis_ = 7.35e-2 μm^2^. As expected, the top panel is just for one representation that values are only 7.35e-2 μm^2^ or 1.125 μm^2^. Adding more representations and averaging over them produces a second peak close to the *P*_c_ value (here 0.7). This is true for any value of *P*_c_. As a result, the fixed and averaged random cases are similar to the extreme case of percolation with *P*_c_ = 1. The third peak appearing in Figure 3 has value close to *P*_c_*K*_sin_.

Zonation effects in the lobule have been treated here by simply varying the spatial expression of the active CYP enzyme. Thus hepactocytes are assumed to be located throughout the lobule tissue as in the Base case model, but the expression of CYP is assumed to be regionally-biased at three levels (zero-, mid-, and full-expression). Two extremes are explored, with the zero expression zone located upstream (i.e. surrounding the arteriole injection site) or downstream (i.e. surrounding the venuole production site). Recall that the Base case model assumed full CYP expression in all hepatocytes of the lobule.

The simulations were performed using the STARS advanced process simulator [15] designed by the Computer Modelling Group (CMG) Ltd. in Calgary, Alberta, to model the flow and reactions of multiphase, multicomponent fluids through porous media [16,17]. Specific biomedical applications of STARS include modeling reactive flow processes in cortical bone [18-21] and in the intervertabral disk [22].

## Results

### Tissue permeability sensitivities on drug transport

Table 1 of the first paper proposed three levels of tissue porosity and permeability, based on a theoretical model of flow and the Carmen-Kozeny equation. The Base case model used the intermediate level for these parameters. Although not shown, we note that the steady state flow rate is basically independent of the tissue permeability level (Ideal, Base, or ECM) as the overall flow is determined primarily by the connected sinusoidal permeability value, which is the same in all three cases.

**Table 1:** Geometric average ranking of random permeability lattices

**Parameter**	**SS Flow Rate (cm**^**3**^**/min)**	**Geometric Average Rate (cm**^**3**^**/min)**
Try1	1.68366e-6	1.9009e-6
Try2	1.71368e-6	1.9246e-6
Try3	1.62196e-6	1.9568e-6
Try4	1.94859e-6	1.9554e-6
Try5	1.99852e-6	1.9891e-6
Try6	1.70890e-6	1.9119e-6
Try7	1.09153e-6	1.9924e-6
Try8	1.34926e-6	1.9038e-6
Try9	1.61186e-6	1.9246e-6
Try10	1.10223e-6	1.9148e-6
Try11	1.21848e-6	1.9365e-6
Base Case	2.44415e-6	2.4415e-6

**Figure 4:**
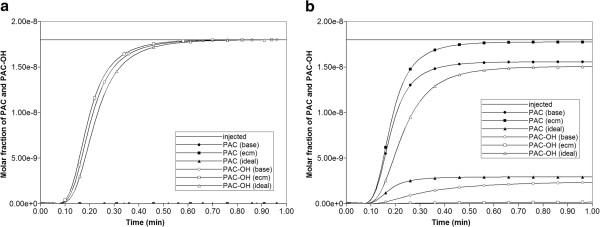
**Reactive (6e-3 min**^**-1**^**) PAC and PAC-OH drug propagation across the lobule, comparing Base, Ideal, and ECM cases (a) with diffusion and (b) without diffusion.**

Figure 4 compares the produced paclitaxol drug profiles versus time for all three tissue permeability levels, with and without additional diffusive transport, respectively. Note that PAC represents the intact drug molecule, and PAC-OH is the metabolite molecule. Paralleling the results in our first paper, the “no diffusion” runs (Figure 4b) are much more responsive to structural changes than the “diffusion” runs (Figure 4a). Diffusion as a process appears to smooth out the structural changes caused by permeability variations, which is a convective effect. When interpreting these results, small-sized drugs may be represented by the diffusive case, while large sized drugs can be viewed as non-diffusive.

**Figure 5:**
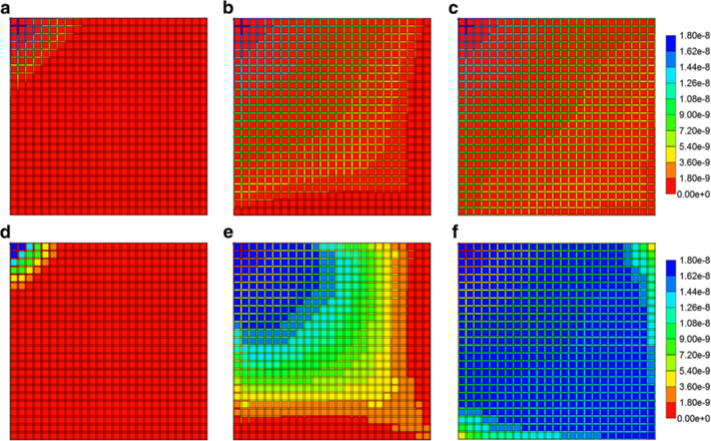
(a) Water production rates, and (b) Non-reactive PAC production profiles for 11 realizations of random permeability lattice.

**Figure 6:**
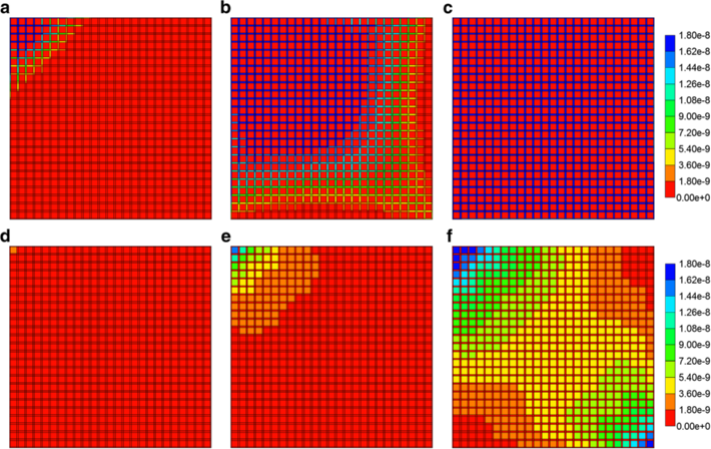
**Reactive (6e-3 min**^**-1**^**) PAC and PAC-OH profiles across the lobule with no diffusion effects for Ideal case metabolism.** PAC at **(a)** 0.01 min, **(b)** 0.14 min and **(c)** 0.50 min; PAC-OH at **(d)** 0.01 min, **(e)** 0.14 min and **(f)** 0.50 min. Color bar is in molfrac.

**Figure 7:**
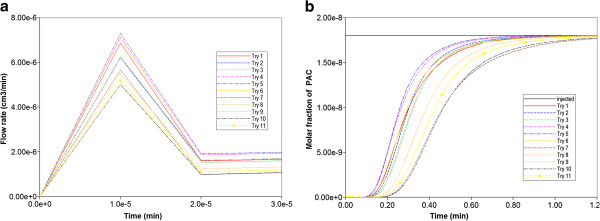
**Reactive (6e-3 min**^**-1**^**) PAC and PAC-OH profiles across the lobule with no diffusion effects for ECM case metabolism.** PAC at **(a)** 0.01 min, **(b)** 0.14 min and **(c)** 0.50 min; PAC-OH at **(d)** 0.01 min, **(e)** 0.14 min and **(f)** 0.50 min. Color bar is in molfrac.

As the component (PAC/PAC-OH) spatial distributions for the flows with diffusion do not vary significantly from those found in the first paper for the Base case tissue permeability (Figure 5 in Paper I), here we discuss only the effect of tissue permeability for reactive flows without diffusion. Figures 6a-f show both PAC and PAC-OH distributions in the Ideal permeability tissue model at 0.01, 0.14 and 0.5 min, respectively. Compared to the Base case runs of the first paper, increasing the tissue permeability to values comparable with the sinusoid permeability levels results in early time conversion of PAC to PAC-OH and an almost uniform distribution of PAC-OH throughout the lobule tissue at later times. In contrast, the more reduced tissue permeability for ECM case results in a much lower and delayed PAC-OH metabolite appearance in the tissue and almost negligible concentration levels in the sinusoids. This is shown in Figures 7a-f for the same three time levels.

### Sinusoid random permeability sensitivities on drug transport

Effects of sinusoidal permeability on paclitaxol drug transport can be explored in the same way as those of tissue permeability presented in the above section. Specifically, representative upper and lower sinusoid permeability values could be selected around the Base case value (*K*_sin_ = 1.125 μm^2^), and their effects on drug transport could be predicted. Such an approach could also illustrate the effects of angio-sensitive molecules causing uniform vascular restriction or dilation of sinusoidal pathways.

**Figure 8:**
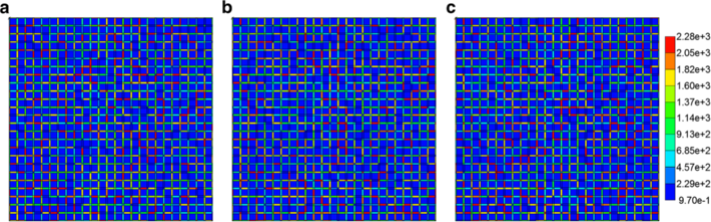
**Generated permeability distribution for (a) 10% (Try11) case, (b) 50% (Try2) case and (c) 90% (Try4) case.** Color bar is in μm^2^.

However, here we examine an alternate, more powerful and representative analysis of sinusoid variability by creating various realizations from an appropriate sinusoid permeability distribution function, as described in the “Methods” section above. Figure 5 illustrates the different production behaviours produced by 11 realizations drawn from a uniform sinusoid permeability distribution with a wide spread (σ = 1) of permeabilities. Figure 5a demonstrates the range of lobule flow rates generated from these permeability distributions produced with a fixed fluid pressure difference across the lobule Δ*p*, while Figure 5b shows the corresponding paxlitaxol production history for each representation. These figures demonstrate that higher flow rates generate earlier paclitaxol production. This method also allows an assessment of the spread of expected behavior by focusing on the 10%, 50%, and 90% cases. For the 11 realizations studied here, these three cases correspond to Try11, Try2, and Try4, respectively. Figures 8a-c show the permeability distribution profiles generated by our algorithm for these three cases.

**Figure 9:**
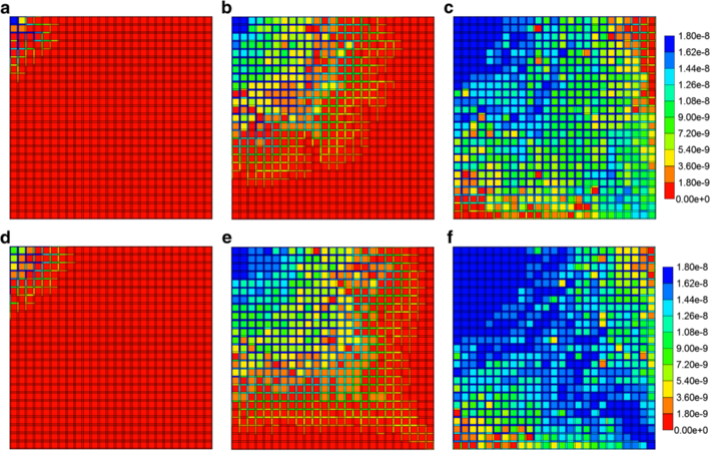
**Non-reactive PAC profile across the lobule for 10% (Try11) case at (a) 0.01 min, (b) 0.14 min, and (c) 0.50 min and for 90% (Try4) case at (d) 0.01 min, (e) 0.14 min, and (f) 0.50 min.** No diffusion effect is considered. Color bar is in molfrac.

Figures 9a-c show non-reactive PAC distributions across the random permeability lobule in the 10% (Try11) tissue realization at 0.01, 0.14 and 0.5 min, respectively, assuming no diffusive contribution to drug flows. This structural variation results in a delayed propagation of the drug across the lobule. In contrast, the increased effective permeability of the 90% (Try4) tissue realization results in a much faster propagation. This is shown in Figures 9d-f for the same three times. The 50% (Try 2) tissue realization results in drug profiles across the lobule visually similar to the 90% realization (not shown explicitly).

The above correlation between observed flow rates and expected paclitaxol production behavior would prove extremely useful in ranking a large number (e.g. 1000) of realizations in terms of extreme behavior, as then only selected simulations need be run to assess paclitaxol production response. We first explored an analytic calculation for effective network permeability, which generated the observed steady state flow rates in Figure 5, via an effective Darcy’s law calculation for a specified Δ*p* as follows:

(1) $$Q_{\mathrm{chs}}=F\;K_{\mathrm{eff}\;}\mathrm{\Delta p}.$$

Here, *F* is a constant network geometric factor that assumes a constant viscosity. For each realization, the effective network permeability can be estimated by a geometric average of all sinusoid and tissue values in a given realization [23] as follows:

(2) $$\ln\left(K_{\mathrm{eff}}\right)=\sum_{i=1}^{N}\frac{\ln\left(K_{i}\right)}{N}.$$

A more general analytic averaging technique, termed power law averaging [24], can also be attempted as:

(3) $$K_{\mathrm{ef}f}^{w}=\sum_{i=1}^{N}\frac{K_{i}^{w}}{N}.$$

Here the power “w” is a best fit parameter. This equation reduces to both arithmetic and harmonic averages when the power “w” is 1 or −1 and becomes the geometric average when “w” is 0.

Finally, an effective permeability calculation that is possibly more correct but less efficient can be generated using the “Effective Medium Theory” [25,26], given in 2D as:

(4) $$K_{\mathrm{ef}f}^{w}=\sum_{i=1}^{N}\left(\frac{K_{\mathrm{ef}\;f}-K_{i}}{K_{\mathrm{ef}\;f}+K_{i}}\right).$$

This requires a root finding method, such as Newton’s method, to find the appropriate answer. Newton’s method requires an initial estimate, appropriately chosen here as the geometric average. Here, we investigated both the geometric and power law averaging techniques, but with limited success.

As an illustration, Table 1 summarizes the geometric mean ranking of the 11 realizations used to generate the drug production responses of Figure 5. Table 1 additionally quotes the flow rate obtained from our Base case (constant sinusoid permeability) model in our first paper. This rate is significantly higher than the flows predicted from any realization, even though individual permeability values were drawn symmetrically from a distribution around the same average permeability. This illustrates that flow paths within the lobule are at least partly in series, especially because of the diverging/converging flow geometry of the model, and hence a harmonic component of flow resistances can be expected (i.e. any lower permeability elements will tend to impart an overall higher resistance to flow).

Eventually we settled on a steady state numerical method to rank realizations. With this approach, we simulated one timestep (with a value large enough to represent steady flow-1.0e-4 min or larger, see Figure 5) of a non-reactive case for each realization. Our (fully implicit) numerical method is extremely stable to such large timesteps and allows the calculation. This would represent one fixed time point for each realization from Figure 5, for ranking purposes.

Rather than the 11 realizations used here for practicality, a more statistically consistent sampling set would include, for example, 101 or 1001 realizations. Utilizing the same methodology of ranking realizations, the 10%, 50%, and 90% cases can again be selected for further detailed analysis. This is the recommended method for analyzing statistical realizations.

### Sinusoid percolation connectivity sensitivities on drug transport

More extreme disturbances in lobule flow conductivity can occur as connectivity is complete (i.e. flow paths are connected). This may be expected to occur with more severe liver damage and disease, such as hepatitis and increased fibrosis. Mathematically, such instances can be appropriately analyzed via percolation theory, with extreme effects seen as the percolation limit is reached.

**Figure 10:**
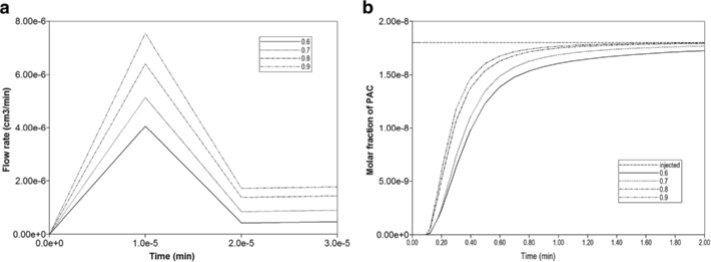
(a) Water flow rates and (b) PAC production rates at various percolation levels.

Figure 10 illustrates the change in flow and non-reactive drug propagation as the percolation value is decreased from *P*_c_ = 1 on the Base case lattice. One sees a trend of decreasing flow and later drug production as the *P*_c_ value is reduced. Each curve represents an average of 100 realizations at a specific percolation value. These realizations were generated as described in the Methods section above. Further analysis of percolation behavior at higher *P*_c_ values could be done as with the random permeability treatments; various realizations for a given *P*_c_ level could be ranked, and single realizations corresponding to 10%, 50%, and 90% cumulative probability could be assessed dynamically. However, as the *P*_c_ level is decreased, the use of geometric mean or effective permeability methods becomes less reliable. As the percolation limit is approached (*P*_c_ = 0.5 for a 2D lattice), the average flow decreases to zero, and each realization can fluctuate wildly in predicted flow.

**Figure 11:**
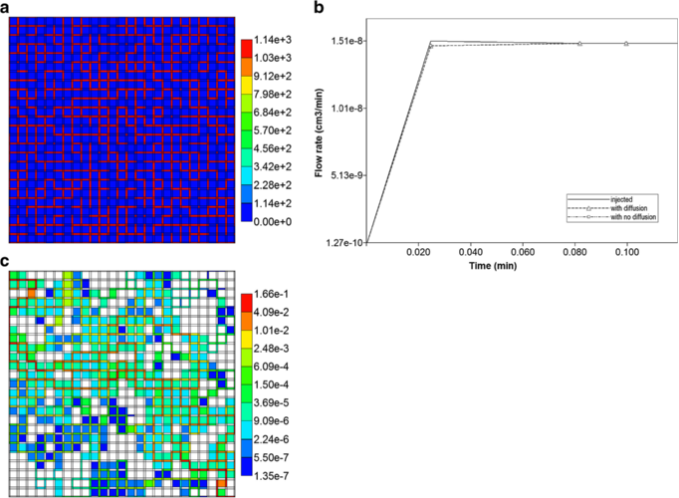
**(a) One realization of a percolation grid at P**_**c**_ = **0**.**55 (color bar is in μm**^**2**^**), (b) injection and production flow rates across one realization of the percolation grid (P**_**c**_ **= 0.55) with or without diffusion and no reaction and (c) total flow velocity profile in cm/min across one realization of the percolation grid (P**_**c**_ **= 0.55).**

Figure 11a shows one realization of a percolation grid at *P*_*c*_ = 0.55. The corresponding flow across this lattice is shown in Figure 11b. It is obvious that the steady state flow rate is 100 times smaller than the Base case level, as is the time to reach steady state. Figure 11c also illustrates the steady state flow velocity distribution across the lobule, showing the many “dead” regions of flow.

Following the analysis of Sauty [3], non-reactive drug propagation across such a lattice can still be expressed as a solution of the convective-dispersion equation with an effective dispersion coefficient, given as

(5) $$C_{R}\left(t_{R},P\right)=0.5\;\mathrm{erfc}\left({\left(\frac{P}{4t_{R}}\right)}^{1/2}•\left(1-t_{R}\right)\right).$$

Here, *P* is the dimensionless Peclet number, expressed as convective flow velocity times length divided by the dispersion coefficient, while *t*_R_ is the dimensionless time, defined as time divided by the time required to get a dimensionless concentration *C*_R_ = 0.5. It should be emphasized that the Sauty solution is for one-dimensional flow while the real flow network here is two-dimensional with the possibility of internal cross flows. For our network, the length of interest is the diagonal distance of 0.106 cm, and the injected concentration is 1.8e-8 (mole fraction), implying a half concentration of 9.0e-9. Fitting the profile should allow estimates of the convective velocity and the effective dispersion for each case.

Table 2 summarizes the best fit results (from MATLAB) to such a profile for four cases–our Base case network model with and without diffusion (see Figure 4 of Paper I), and the percolation realization, also with and without diffusion. Generally, and following the analysis of Levitt [27], one should expect increased effective dispersion coefficients by adding molecular diffusion to the “network dispersion” of the regular lattice and even more “network dispersion” of the percolation lattice. Two calculations of effective dispersion coefficients from the fit value of the number are given. The first column uses the Base case flow velocity for all calculations. Conversely, the second effective dispersion column recognizes the reduced flow velocities occurring in the percolation network and adjusts the effective dispersion coefficient correspondingly. From this perspective, the relative sizes of effective dispersion coefficients for the four cases in Table 2 can be rationalized.

**Table 2:** **Production profile fit to sauty**[3]**analytic convection-diffusion profile**

	**C**_**half**_	**t**_**R**_	**P**	**P**^**-1**^	**K**_**eff**_	**K**_**eff_rs**_	**χ**^**2**^
Reg_nodiff	8.99e-9	0.2194 min	7.656	0.1306	1.306e-3	1.306e-3	1.83e-9
Reg_diff	9.00e-9	0.2228 min	6.503	0.1538	1.538e-3	1.538e-3	1.90e-9
Perc_nodiff	8.56e-9	8.7990 min	4.069	0.2458	2.548e-3	6.370e-5	66.2e-9
Perc_diff	9.02e-9	18.390 min	3.609	0.2771	2.771e-3	6.928e-5	1.11e-9

**Figure 12:**
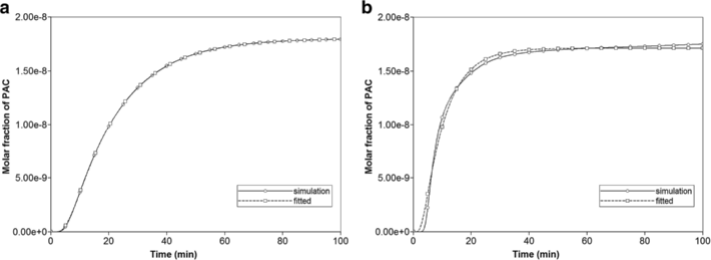
**Production profile fit to Sauty **[3]**analytic convection-diffusion profile for (a) percolation example with diffusion, and (b) percolation example without diffusion.**

Figures 12a, b show the values for the percolation realization, with and without diffusion. For non-reactive flow, percolation effects are seen to significantly reduce drug transit times across the lobule, while generating a “structure-induced” increased effective dispersion coefficient for the produced drug profile. Also, while the matches to the Sauty theoretical profile are excellent for the percolation case with diffusion (and for the regular lattice Base case with and without diffusion), the no-diffusion percolation case fit is poor. Although somewhat unclear from Figure 12b (MATLAB is attempting a “best-fit” to the Sauty profile), this simulated case actually shows a long-time deviation from the simple Sauty profile as the produced concentration approaches the injected value. This can be attributed to slowly diffusing concentrations from partially blocked areas inside the network as the outlet concentration eventually sweeps these areas. Although we did not attempt equivalent fitting of our production profiles from our random permeability realizations to the Sauty profiles, we expect that smaller random deviations from our Base case model will also produce excellent “Sauty profile” fits, while random realizations with more extreme distributions may not.

**Figure 13:**
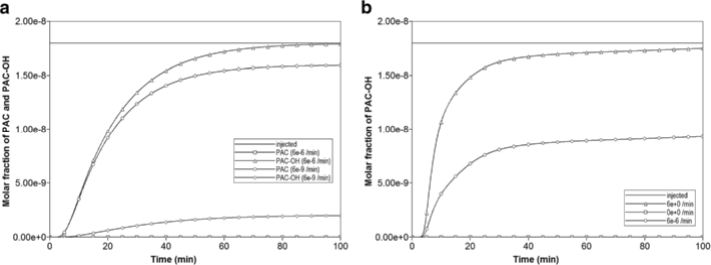
**Reactive PAC-OH profiles across the percolation lobule (P**_**c**_ **= 0.55) (a) with diffusion effects and two metabolic rates, and (b) with no diffusion effects and various metabolic rates.**

Next, we investigated reactive (metabolite-generating) processes on a percolation lobule. Figure 13 shows reactive PAC and PAC-OH production profiles for this realization, at two metabolic rates. With diffusion, PAC-OH is produced at both reaction rates, but at different levels (Figure 13a), while without diffusion, only extremely high reaction rates will produce PAC-OH (Figure 13b).

**Figure 14:**
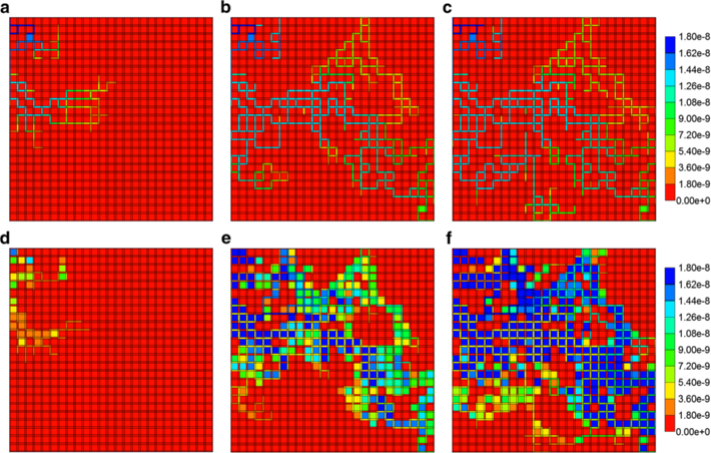
**Reactive (6e-6 min**^**-1**^**) PAC profile across the percolation lobule (P**_**c**_ **= 0.55) (a) at 2 min, (b) at 28 min and (c) at 100 min with no diffusion effect.** Reactive (6e-6 min^-1^) PAC-OH profile across the percolation lobule (P_c_ = 0.55) **(d)** at 2 min, **(e)** at 28 min and **(f)** at 100 min with no diffusion effect. Color bar is in molfrac.

**Figure 15:**
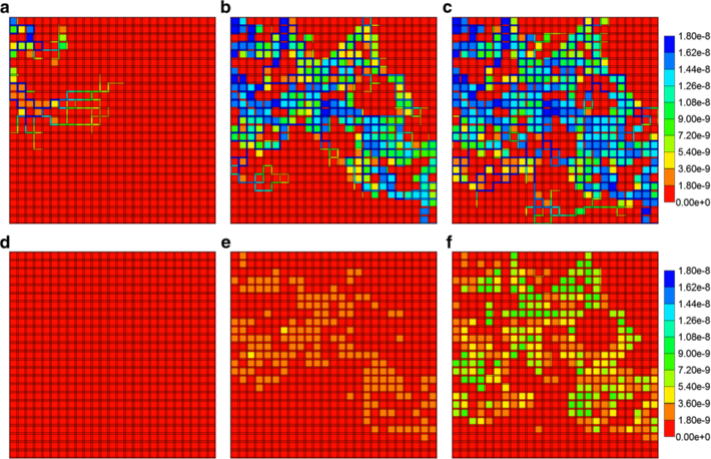
**Reactive (6e-9 min**^**-1**^**) PAC profile across the percolation lobule (P**_**c**_ **= 0.55) (a) at 2 min, (b) at 28 min and (c) at 100 min with no diffusion effect.** Reactive (6e-9 min^-1^) PAC-OH profile across the percolation lobule (P_c_ = 0.55) **(d)** at 2 min, **(e)** at 28 min and **(f)** at 100 min with no diffusion effect. Color bar is in molfrac.

Reactive PAC and PAC-OH profiles across the percolation lobule are shown in Figures 14 and 15. Here again we consider the case neglecting diffusive flow contributions to drug transport. The specified reaction rate in Figures 14a-f corresponds to the Base case rate of our first paper. Here the PAC profiles follow directly the percolation paths through the lobule, while PAC-OH is generated only in the tissue directly surrounding these paths. At a lower metabolic conversion rate, the effects on PAC-OH distribution are even more extreme, as illustrated in Figures 15a-f.

### Zonation and effects on drug elimination

Two zonation cases were analyzed: the upstream (normal) case, where CYP expression is active primarily near the drug inlet zone, and the downstream (reversed) case, where CYP expression is active primarily near the drug outlet zone. Typically, CYP expression is expected to follow the latter case, but both are analyzed for comparison.

**Figure 16:**
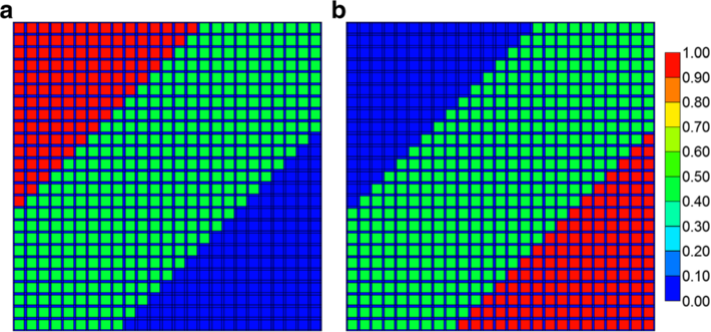
Relative CYP concentration for (a) “extreme zonation” case and (b) “extreme reversed zonation” case.

For each case, two scenarios were envisioned, an averaged (minor) difference in CYP activity in the three zones, and an extreme scenario with larger differences in CYP activity between the three zones (one of which has zero CYP activity, either upstream or downstream). Table 3 lists relative CYP levels for each case. Figures 16a-b lists the assumed CYP expression levels for the two extreme cases.

**Table 3:** Relative CYP levels in ideal zonation cases

**Case**	**Relative CYP Levels**	**(Upstream/Middle/Downstream)**
Average zonation	0.75/0.50/0.25	(U/M/D)
Extreme zonation	1.00/0.50/0.00	(U/M/D)
Average reverse zonation	0.25/0.50/0.75	(U/M/D)
Extreme reverse zonation	0.00/0.50/1.00	(U/M/D)

**Figure 17:**
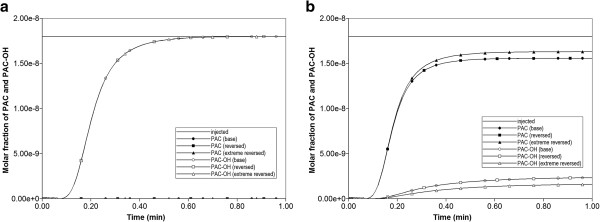
**Reactive (6e-3 min**^**-1**^**) PAC and PAC-OH metabolite production for reversed zonation cases (a) with diffusion and (b) without diffusion.**

The reversed zonation case is considered first. Figure 17a compares reactive PAC and PAC-OH production profiles with diffusion contributions to the flow. Zonation (either averaged or extreme) is seen to have no effect on the production behavior compared to the Base case (no zonation) behavior. Note also that only PAC-OH is produced here. However, with the same (reversed) zonation patterns, but assuming no diffusive contribution to the flow, mostly unmetabolized PAC is produced, and some differences in production behavior are seen with zonation. This is summarized in Figure 17b.

**Figure 18:**
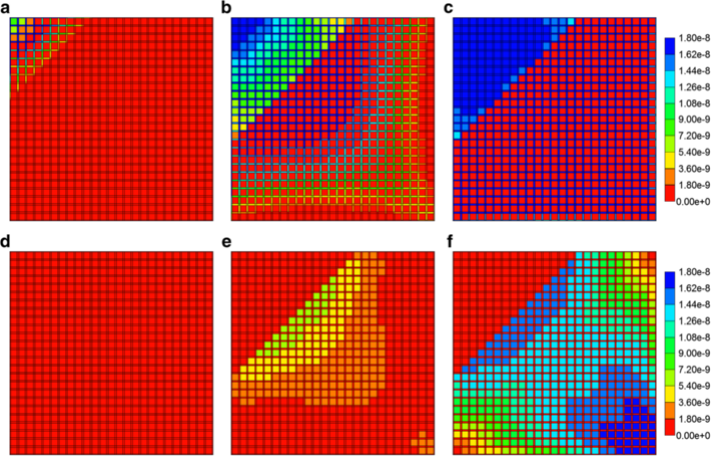
**Reactive (6e-3 min**^**-1**^**) PAC and PAC-OH profiles across lobule for extreme reversed case without diffusion.** PAC at **(a)** 0.01 min, **(b)** 0.14 min and **(c)** 0.50 min; PAC-OH at **(d)** 0.01 min, **(e)** 0.14 min and **(f)** 0.50 min. Color bar is in molfrac.

These observations do not imply that unchanging metabolite distributions are occurring within the lobule itself, as a function of assumed zonation. Figures 18a-f illustrate the PAC and PAC-OH distributions across the lobule at 0.01 min, 0.14 min, and 0.50 min, respectively, for the extreme reversed zonation case without diffusion. A definite upstream effect of no CYP metabolite processing is seen, but the more downsteam levels of CYP can counteract this lack of activity, at least with the reaction rate assumed in these runs. These results should be compared with the Base case (no zonation) runs presented in our first paper. PAC and PAC-OH profiles at the equivalent time points for the same extreme reversed zonation model, but with diffusive flow, are not shown explicitly.

**Figure 19:**
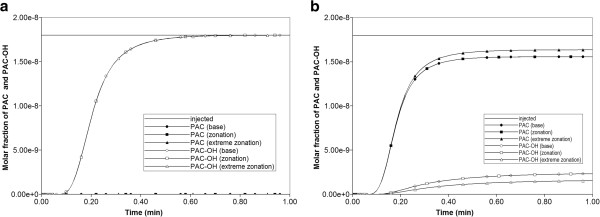
**Reactive (6e-3 min**^**-1**^**) PAC and PAC-OH metabolite production profiles for zonation cases (a) with diffusion and (b) without diffusion.**

The normal zonation case is considered next. Figure 19a compares reactive PAC and PAC-OH production profiles with diffusion contributions to the flow. Zonation (either averaged or extreme) is seen to have no effect on the production behavior compared to the Base case (no zonation) behavior. Note also that only PAC-OH is produced here. However, with the same (normal) zonation patterns, but assuming no diffusive contribution to the flow, mostly unmetabolized PAC is produced, and some differences in production behavior are seen with zonation. This is shown in Figure 19b.

**Figure 20:**
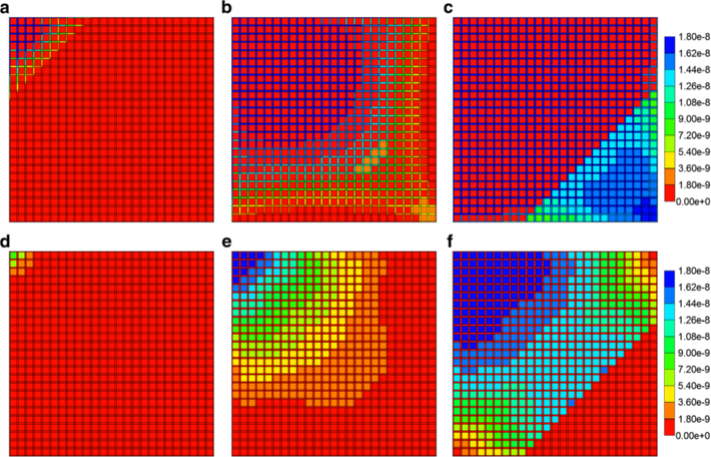
**Reactive (6e-3 min**^**-1**^**) PAC and PAC-OH profiles across lobule for extreme case without diffusion.** PAC at **(a)** 0.01 min, **(b)** 0.14 min and **(c)** 0.50 min; PAC-OH at **(d)** 0.01 min, **(e)** 0.14 min and **(f)** 0.50 min. Color bar is in molfrac.

Zonation again affects the different metabolite distributions within the lobule itself. Figures 20a-f illustrate the PAC and PAC-OH distributions across the lobule at 0.01 min, 0.14 min, and 0.50 min, respectively, for the extreme normal zonation case without diffusion. As discussed above, a definite upstream effect of no CYP metabolite processing is seen, but the more downsteam levels of CYP can counteract this lack of activity, at least with the reaction rate assumed in these runs. These results should be also compared with the Base case (no zonation) runs presented in our first paper. PAC and PAC-OH profiles at the equivalent time points for the same extreme normal zonation model, but with diffusive flow are not shown explicitly.

### Fractal behaviour in the liver-an alternate perspective

Previously, to take into account organ heterogeneity and simulate enzyme kinetics in disordered media, lattice models have been introduced by several investigators. Berry [28] performed Monte Carlo simulations of a Michaelis-Menten reaction on a two-dimensional lattice with a varying density of obstacles to simulate the barriers to diffusion caused by biological membranes. He found that fractal kinetics resulted at high obstacle concentrations. Kosmidis et. Al. [29] performed Monte Carlo simulations of a Michaelis-Menten enzymatic reaction on a two-dimensional percolation lattice at criticality. They found that fractal kinetics emerged at large times.

Previously [30], we developed a network model of the liver consisting of a square lattice of vascular bonds connecting two types of sites that represent either sinusoids or hepatocytes. Random walkers with a drift velocity explored the lattice and were removed with a set probability from hepatocyte sites. To simulate different pathological states of the liver, random sinusoid or hepatocyte sites were removed. For a lattice with regular geometry, it was found that the number of walkers decayed according to an exponential relationship. For a percolation lattice with a fraction *p* of the bonds removed, the decay was found to be exponential for high trap concentrations but transitioned to a stretched exponential at low trap concentrations.

These models are all basically random walk models (emphasizing diffusive flow), and the lattices are abstract representations of the geometry of the space. Here we have created network models that incorporate realistic anatomical and physiological properties of the liver as well as emphasizing the consequent convective flow behavior of the well-perfused liver lobule. Here the fractal behavior of the liver lobule results from flow inhomogeneities.

**Figure 21:**
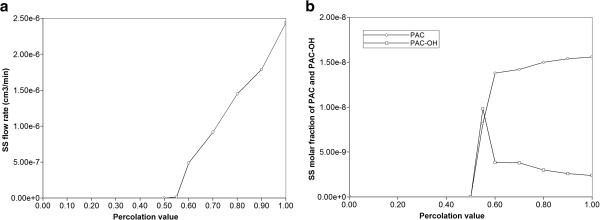
Percolation Network effects on (a) Steady State Flow (b) Produced Steady State Reactive PAC and PAC-OH without diffusion.

Figure 21 illustrates the reactive flow behavior as a function of percolation value, based on our percolation lattice models described earlier. Data analogous to Figure 10, but for base case reactive flow parameters, were used to generate these plots. Figure 21a shows a decreasing steady state flow pattern as the lattice percolation value is decreased to the 2D percolation limit of 0.5. This is analogous to the percolation patterns on 2D lattices observed many years ago by Kirkpatrick [25] (see his Figure 3). Here, Figure 21b illustrates the role of percolation on steady state produced PAC and PAC-OH as a function of percolation value, using the Base case reaction parameters. These illustrate the role of incomplete mixing on reactive behavior in a normally convective-dominated network, especially as the percolation limit is approached (and where convection ceases to be so dominant). At or below the percolation threshold of 0.5, no PAC or PAC-OH is produced.

## Conclusion

Together with our previous paper, this work presents a useful framework for analyzing the coupled and competing flow processes (convection/diffusion/reaction) that determine drug propagation and availability in a well-vascularized tissue such as a liver lobule. With our numerical approach, we have addressed both multidimensional spatial aspects as well as transient and steady state time behavior. In this paper, we have emphasized the impact of structural variability (including enzyme zonation) on non-uniform spatial distributions of drug and drug-metabolites occurring across the lobule. In particular, our non-dispersive models are shown to be more sensitive to such structural variations. We have also illustrated several techniques to quantify and analyze the role of such spatial variability.

Our network models including dispersive effects often correspond to the “well-stirred” compartment models [31], such that relatively uniform steady-state concentration levels occur throughout the lobule (if one ignores the smaller inlet mixing zone). Conversely, simulations on our network models without explicit dispersive mixing often correspond to modified “parallel tube” models [32], in which observed concentration profiles change along the length of the tubes (i.e. sinusoids). (Here our modified tube network structure allows cross-sinusoidal flow as well).

Aging and liver cirrhosis have been found to effect the transfer of drugs and metabolites from sinusoids to tissue, rather than directly effect the intrinsic metabolic process of hepatocytes [33,34]. Recognizing that the dispersive mixing terms in our models allow easy permeation across the sinusoid-tissue interface, as well as improved intracellular transport, our calculated concentration profiles “with diffusion” can be viewed as representative of healthy liver behavior, while our non-diffusive profiles can be interpreted as representing aged or cirrhotic liver behavior.

A subsequent paper will expand this analysis to include sensitivities associated with variations in realistic lobule structure obtained from lobule images, which could even more realistically reflect extents of liver damage.

## Competing interests

The authors declare that they have no competing interests.

## Authors’ contributions

VR: Primary investigator who conducted the majority of the simulations and model development. RM: Initial investigator who conducted the preliminary simulations and model development. DC: Investigator providing technical support for the simulations and model development. JT: Lead investigator who supervised the work content and model development. All authors read and approved the final manuscript.
